# Adipocytokines in Atherothrombosis: Focus on Platelets and Vascular Smooth Muscle Cells

**DOI:** 10.1155/2010/174341

**Published:** 2010-06-28

**Authors:** Giovanni Anfossi, Isabella Russo, Gabriella Doronzo, Alice Pomero, Mariella Trovati

**Affiliations:** Department of Clinical and Biological Sciences, Internal Medicine and Metabolic Disease Unit, San Luigi Gonzaga Hospital, San Luigi Gonzaga Faculty of Medicine of the Turin University, Orbassano,10043 Turin, Italy

## Abstract

Visceral obesity is a relevant pathological condition closely associated with high risk of atherosclerotic vascular disease including myocardial infarction and stroke. The increased vascular risk is related also to peculiar dysfunction in the endocrine activity of adipose tissue responsible of vascular impairment (including endothelial dysfunction), prothrombotic tendency, and low-grade chronic inflammation. In particular, increased synthesis and release of different cytokines, including interleukins and tumor necrosis factor-*α* (TNF-*α*), and adipokines—such as leptin—have been reported as associated with future cardiovascular events. Since vascular cell dysfunction plays a major role in the atherothrombotic complications in central obesity, this paper aims at focusing, in particular, on the relationship between platelets and vascular smooth muscle cells, and the impaired secretory pattern of adipose tissue.

## 1. Introduction

Subjects affected by central obesity (i.e., by intra-abdominal fat excess) are characterized by insulin resistance, metabolic disorders, and vascular abnormalities which cooperate to induce high cardiovascular risk [1–3]. 

The so-called “metabolic syndrome”, defined on the basis of a combination of central obesity, glucose intolerance, atherogenic dyslipidemia, and arterial hypertension [3, 4], is present in the large majority of these subjects. 

In central obesity, abnormalities in the extent and distribution of fat mass are associated with a peculiar dysfunction of adipose tissue, responsible—together with the insulin resistance—of alterations of vascular function (including endothelial dysfunction), pro-thrombotic tendency, low-grade chronic inflammation, and oxidative stress: these defects, frequently associated as a cluster, represent the main pathogenetic link between obesity and the increased risk of athero-thrombotic events [5–7].

Omental adipose tissue, which comprises both adipocytes and a stromovascular cell fraction, is not an inert lipid storage site, but a dynamic endocrine organ able to synthesize and secrete many bioactive peptides—collectively named “adipocytokines”—deeply involved in the metabolic, vascular, and immunological homeostasis by paracrine and endocrine mechanisms [2, 6, 8–10].

Some molecules, directly synthesized by adipocytes and called “adipokines” (i) control energy balance and appetite, and influence insulin sensitivity via endocrine mechanisms, and (ii) modulate adipocyte size/number and angiogenesis via paracrine mechanisms, thus exerting a major role in the regulation of fat mass [10, 11]. Furthermore, they can also exert a role in the control of blood pressure, lipoprotein metabolism, coagulation, immunity and inflammation [5, 11]. 

Other peptides belonging to the cytokine group—produced and released by the stromal vascular components of adipose tissue (i.e., lymphocytes, fibroblasts, macrophages, endothelial cells, and preadipocytes) [8, 9, 12–14]—are mainly involved in local and systemic inflammation [8, 13–16]. The increase in abdominal adipose tissue mass dysregulates both adipokine and adipocytokine secretion patterns [10, 14]. 

With the exception of the insulin sensitizing peptide adiponectin, adipokine production and secretion are increased in central obesity [10, 11, 15]: this fact plays a pivotal role both in the pathogenesis of cardiovascular damage through adverse effects on hemostatic balance and vascular function [5, 6, 11], and in the amplification of inflammatory processes in vascular and nonvascular tissues [11, 13, 15]. 

Also, cytokine release is enhanced: this phenomenon is attributable to increased prevalence of hypertrophied adipocytes with altered adipokine synthesis and secretion, local hypoxia, as well as activation of resident inflammatory cells and macrophages [14, 15]. 

In particular, adipose tissue from individuals with central obesity synthesizes and releases increased amount of

- proinflammatory chemokines and cytokines, such as Monocyte Chemoattractant Protein-1 (MCP-1), macrophage migration inhibitory factor (MIF), tumor necrosis factor-*α* (TNF-*α*), and interleukins, including interleukin-1*β* (IL-1*β*) and interleukin-6 (IL-6) [15];
- procoagulant and proinflammatory mediators such as tissue factor (TF) and plasminogen activator inhibitor-1 (PAI-1) [15];
- vasoactive substances such as angiotensinogen and endothelin-1 (ET-1) [17, 18];
- molecules involved in the pathogenesis of insulin resistance, such as TNF-*α* and resistin [8, 10, 13–16].

Since central obesity is characterized by an enhanced cardiovascular risk, and it is known that dysfunctions of platelets and vascular smooth muscle cells (VSMC) are deeply involved in athero-thrombosis [19–22], the purpose of this review is to identify the actions of adipokines and adipocytokines on these cells, focusing in particular on the role of inflammation.

## 2. Role of Platelets in Thrombosis and Inflammation

Platelets are anuclear cell fragments released from megakaryocytes, hematopoietic cells that differentiate and undergo endomitosis [23, 24]. Despite the lack of nucleus, they contain mRNA and spliceosomal components for mRNA processing, as well as the translational machinery for protein synthesis [23, 25]: this justifies *de novo* synthesis by platelets of different mediators involved in the regulation of inflammatory and coagulant pathways including IL-1*β*, PAI-1, and TF [26, 27].

**Figure 1:**
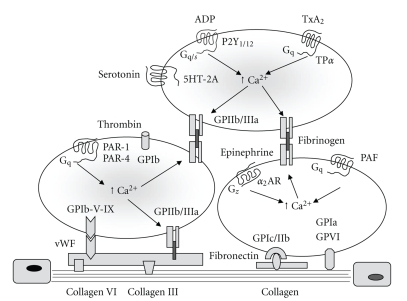
Mechanisms involved in platelet adhesion to subendothelial layer and activation. The diagram illustrates the role of von Willebrand factor (vWF), collagen and other proteins in platelet adhesion by linking to exposure of membrane glycoprotein receptors including GPIb//V/IX complex, GPIIb/IIIa, and GPVI which ensures a stable anchorage with subendothelial matrix by interaction with collagen. Platelet activation and aggregation are triggered by thrombin, endogenous mediators released from storage granules, and synthesis of platelet activating factor (PAF), and thromboxane A_2_ (TXA_2_). P2Y1/12, purinergic P2Y receptors; TP*α*, thromboxane *α* receptor; 5HT-2A, serotonin (5-hydroxytryptamine)-2A receptor; PAR-1, protease-activated receptor-1; PAR-4, protease-activated receptor-4; *α*_2_ AR, *α*_2_ adrenoreceptor.

As evidenced in Figure 1, platelets play a pivotal role in the response to vascular injury through adhesion to exposed subendothelial layer triggered by different collagen types and adhesive proteins such as von Willebrand factor (vWF), fibronectin, laminin, fibulin and thrombospondin [26–29]. Activation of platelets by components of the subendothelial matrix is linked to exposure of membrane glycoprotein receptors including GPIb/V/IX complex which interacts with vWF, integrin *α* IIb*β*_3_ (GPIIb/IIIa) able to bind Arg-Gly-Asp [RDG] domain of vWF and fibrinogen, and GPVI which ensures a stable anchorage with subendothelial matrix by direct interaction with collagen [26–30]. Platelet activation and formation of aggregates are triggered also by thrombin [31], endogenous mediators released from storage granules and erythrocytes such as adenosine 5-diphosphate (ADP), and *de novo* synthesis of platelet activating factor (PAF), and thromboxane A_2_ (TXA_2_) [30, 32–34].

**Figure 2:**
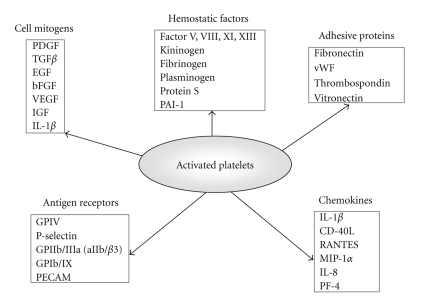
Platelet components involved in the coagulation cascade and the atherosclerotic process. PDGF, platelet-derived growth factor; TGF*β*, transforming growth factor *β*; EGF, endothelial growth factor; bFGF, fibroblast growth factor; VEGF, vascular endothelial growth factor; IGF, insulin-like growth factor; IL-1*β*, interleukin-1*β*; PAI-1, plasminogen activator inhibitor 1; vWF, von Willebrand factor; GP, glycoproteins; PECAM, platelet and endothelial cell adhesion molecule; CD40L, CD40 ligand (CD154); RANTES, regulated on activation, normal T-cell expressed and secreted; MIP-1*α*, macrophage inflammation protein 1*α*; IL-8, interleukin-8; PF4, platelet factor 4.

As shown in Figure 2, activated platelets also release inflammatory mediators from granules, including platelet-derived growth factor (PDGF) and platelet factor 4 (PF-4) [35–37].

Beyond acute activation as a consequence of vascular injury, circulating platelets are actively involved in all phases of the atherogenetic process, from atherosclerotic plaque formation to plaque inflammation and rupture [28, 37–39]. In these conditions, platelet reactivity is increased by reactive oxygen species (ROS) produced as a consequence of oxidative stress, by reduction of endothelial antithrombotic properties and by the increased availability of proinflammatory mediators, such as cytokines and chemokines [40, 41].

Actually, platelets release several mediators linking thrombosis and vascular inflammation such as the RANTES (regulated on activation, normal T-cell expressed and secreted) chemokine, PDGF, PF-4, Transforming growth factor-*β* (TGF-*β*), CD40 ligand (CD40L, CD154), P-selectin, and TXA_2_ [35–37, 41].

RANTES recruits monocytes and T cells; in conjunction with P-selectin this chemokine can be immobilized on inflammed endothelial cells, thus inducing monocyte arrest and migration [41].

PDGF, the major growth factor contained in platelets [36–38], stimulates both migration and proliferation of VSMC by co-operation with serotonin, and TGF-*β* [36, 42, 43], and is chemotactic for monocytes [38, 43]; its effects on VSMC are critical for the development of atherosclerotic process [27, 37, 38].

PF-4—a member of the C-X-C chemokine subfamily—exerts chemotactic effects on monocytes, promotes monocyte-to-foam cell differentiation [27, 35, 36], enhances the binding of oxidized low density lipoproteins (LDL) to vascular wall, and inhibits LDL degradation through the LDL receptors [35, 44].

CD40L is a trimeric protein structurally related to TNF-*α* superfamily stored in the *α* -granules of resting platelets [45–49]. After platelet activation, it is rapidly exposed on cell surface and cleaved to release the soluble fragment (sCD40L) able to increase the stability of new platelet aggregates [49], and to activate vascular inflammatory mechanisms by inducing production of ROS, enhancing expression of adhesion molecules Vascular Cell Adhesion Molecule-1 (VCAM-1), Intercellular Adhesion Molecule-1 (ICAM-1), and E-selectin in endothelial cells and VSMC, and increasing secretion of cytokines, chemokines, matrix metalloproteinases (MMPs), and procoagulant factors [45–48]. 

Several cell types—including endothelial cells, VSMC, monocytes, neutrophils, B cells and fibroblasts—bind CD40L through the specific receptor CD40 [45, 50]. This receptor has been recently identified also in adipocytes where it plays a relevant role in the crosstalk with resident lymphocytes [51]; furthermore, circulating sCD40L levels have been found at abnormally high levels in patients with obesity, type 2 diabetes mellitus and atherosclerotic vascular diseases [52–55].

P-selectin is stored in platelet *α* -granules [36] and, after activation, rapidly translocated upon cell surface becoming accessible to circulation; this phenomenon strengthens initial rolling contact between platelets and vessel wall and promotes RANTES deposition on endothelial cells, thus increasing monocyte recruitment [27–29, 41, 47, 56].

**Figure 3:**
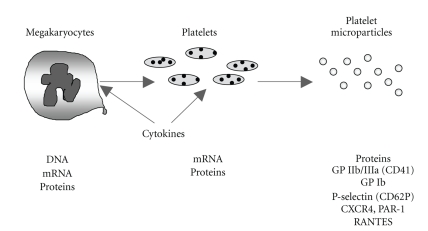
General features of circulating platelet microparticles (PMPs). PMPs are phospholipid microvesicles of 0.1–1 micron in size, sheded from parental cell fragments after stimulation with physiological agonists such as thrombin or collagen or exposure to shear stress (i.e., in severe stenosis). PMPs express functional adhesion receptors, including GPIIb/IIIa (CD41), P-selectin (CD62P), CXCR4, and PAR-1 and contain different proteins and coagulation factors, thus exerting a role in the hemostatic response and in the interplay between coagulation and inflammation. PMPs also simulate cytokine release and adhesion molecule expression in endothelial cells and contraction of VSMC. Elevated levels of circulating PMPs have been described in patients with arteriosclerosis, acute vascular syndromes, diabetes mellitus, as well as central obesity. GPIIb/IIIa, glycoprotein IIb/IIIa; GP Ib, glycoprotein Ib; CXCR4, chemokine (C-X-C motif) receptor 4; PAR-1, protease-activated receptor-1; RANTES, regulated on activation, normal T-cell expressed and secreted.

 Platelet stimulation by agonists or exposure to high shear stress leads to formation and release of platelet-derived membrane-coated vesicles termed platelet microparticles (PMPs) [57, 58], which influence the activities of other cell types both regionally and systemically [58]. As evidenced in Figure 3, PMPs are lipid-protein complexes with a diameter < 1 *μ* m, composed by vesicular fragments of the plasma membrane and *α* -granules [59]: their protein content plays a relevant role in both hemostasis and inflammation, by facilitating coagulation, promoting platelet and leukocyte adhesion to the subendothelial matrix, supporting angiogenesis and stimulating VSMC [58–60]. These effects may contribute to the chronic inflammatory state which characterizes atherosclerosis [58]; in particular, a portion of platelet-derived IL-1*β* associated with PMPs stimulates the production of chemoattractant molecules and cytokines and the expression of specific adhesion molecules in endothelial cells, thus enhancing their interaction with circulating leukocytes and, therefore, their ability to trigger inflammatory responses [61, 62]. Also the chemokine RANTES is delivered to sites of endothelial injury via PMPs to promote monocyte recruitment [56, 63].

## 3. Alterations of the Platelet Function in Central Obesity

Platelet hyperactivity is deeply involved in the increased atherothrombotic risk of patients affected by central obesity and type 2 diabetes mellitus [20, 21, 64]. 

A variety of defects of platelet function—mainly related to increased adhesiveness and activability* in vivo* and reduced sensitivity to physiological antagonists—has been identified in central obesity, as recently reviewed [20, 21]. 

Mean volume of circulating platelets—a parameter directly related to *in vivo* platelet activation [65] relevant to predict myocardial infarction occurrence and mortality and restenosis following coronary angioplasty [66]—is increased in obese patients, independently of the presence of other cardiovascular risk factors [66, 67]; furthermore, a positive correlation between body mass index (BMI) and mean platelet volume (MPV) has been found in obese individuals [66–69], whereas weight loss may lead to a decrease in platelet size and reactivity [68].

Increasing evidences indicate that the size of circulating platelets deeply influences their hemostatic potential being the response of larger platelets to aggregating stimuli more rapid and the amount of released mediators increased [65, 70].

Platelet volume, as well as other platelet parameters, are mainly determinated during megakaryocyte fragmentation in bone marrow [71, 72]. Even though the mechanisms influencing magakaryocytopoiesis are not completely understood, an involvement of inflammatory cytokines (including interleukins -1, -3, -6, -8, -11, and -18), and of nitric oxide (NO) has been shown in some studies [73, 74]; this phenomenon allowed to hypothesize that the increased production and release of proinflammatory cytokines, as well as endothelial dysfunction of central obesity, might influence megakaryocytopoiesis and circulating platelet volume [75]. 

The increased *in vivo* activation of circulating platelets is mirrored by the enhanced expression of activation-dependent adhesion molecules, and by increased plasma concentrations of sP-selectin; another index of *in vivo* platelet activation—that is, urinary excretion of 11-dehydro-TXB_2_, the major enzymatic metabolite of TXA_2_—is increased in women affected by visceral obesity, compared to non-obese women [76].

Relevant defects of platelet function in central obesity are related also to a reduced sensitivity to mediators—such as insulin, prostacyclin (PGI_2_), and NO—which in lean subjects reduce platelet sensitivity to proaggregating stimuli [21]. 

Insulin, which physiologically reduces platelet responses to agonists both *in vitro* [77–79] and *in vivo *[77, 80, 81], mainly through a NO-dependent mechanism mediated by the increase of intraplatelet cyclic nucleotides 3′, 5′ -cyclic guanosine monophosphate (cGMP) and 3′, 5′ -cyclic adenosine monophosphate (cAMP) [78, 82], exhibits a deeply impaired antiaggregating effect in insulin resistant states, such as central obesity, type 2 diabetes mellitus with obesity and essential arterial hypertension [81, 83–85].

Furthermore, platelets from obese subjects or obese type 2 diabetic patients show defective responses to the NO/cyclic nucleotide/protein kinase pathway including the ability of NO and NO donors to increase cGMP, the ability of cGMP to reduce platelet calcium and consequently aggregation [86, 87]; similarly, also the ability of PGI_2_ to increase cAMP and of cAMP to reduce platelet function are impaired in these patients [87].

As previously mentioned, a relevant factor causing plaletet dysfunction is the increased oxidative stress [40], which is present in central obesity, as a consequence of imbalance between ROS production and reduced levels of substances able to protect from the damage of free radicals and peroxides [88]. Several metabolic abnormalities of the patients with visceral obesity—that is, excess of circulating free fatty acids, of oxidized LDL, and of proinflammatory adipokines and cytokines—contribute to ROS production [88, 89].

Some adipokines and inflammatory cytokines such as TNF-*α* and leptin are involved in enhanced oxidative stress [88, 89]; furthermore, the increase in fat mass, as well as the pathological secretion pattern of adipocytes decrease the availability of antiinflammatory proteins such as adiponectin and ghrelin, which exert also protective effects against oxidative stress [15, 90, 91].

High concentrations of ROS influence platelet function by different mechanisms, including decreased NO bioavailability, calcium mobilization abnormalities and over-expression of membrane glycoproteins [40, 92–94].

Isoprostanes are a family of prostaglandin-like metabolites produced *in vivo* from esterified arachidonic acid of cell membrane phospholipids or lipoproteins [95, 96] through a ROS-dependent mechanism [96–99], completely independent of cyclo-oxygenase-1 (COX-1) activity [95–97]. Once released, they circulate in plasma and are available for receptorial interaction with platelets and cells of the vascular wall; in particular, 8-iso-prostaglandin-*F*_2*α*_ (8-iso-*P* *G* *F*_2*α*_), which is an abundant isoprostane compound formed *in vivo* in humans, induces vasoconstriction and amplifies the adhesive reactions and the aggregating responses of human platelets to agonists [100, 101].

Several studies showed a stimulatory interaction of isoprostanes with TXA_2_ receptors [102, 103], which is prevented by TXA_2_ receptor antagonism but not by COX-1 inhibition [99]. Recently, a further isoprostane binding site, responsible of cAMP reduction, has been recognized [103]. 

The increased levels of F2-isoprostanes observed in visceral obesity [104–107] can be involved both in the persistent platelet activation *in vivo* [104], and in the resistance to antiplatelet effects of aspirin [21].

## 4. Alterations of the Vascular Smooth Muscle Cells in Obesity

### 4.1. Role of Vascular Smooth Muscle Cells in Physiological Conditions

In physiological conditions VSMC play an essential role in providing structural integrity of the vessel wall and in controlling vascular tone and blood pressure [108, 109]; in particular, this cell type is the main target of the effects of endothelium-released NO, which stimulates the synthesis of cGMP, thus preventing the calcium release from intracellular stores [110, 111]. The inhibition of calcium-dependent Rho/Rho kinase pathway is a relevant mechanism involved in the modulation of VSMC relaxation induced by the NO/cGMP/PKG pathway [112]. 

The surface complex system which regulates VSMC responses and modulates the contractile process involves the expression of receptors for catecholamines, acetylcholine, serotonin, histamine, purinergic mediators, angiotensin II (Ang II), bradykinin, neuropeptide Y, Vasoactive Intestinal Polypeptide (VIP), vasopressin, oxytocin, prostanoids, leukotrienes, and growth factors [PDGF, Epidermal Growth Factor (EGF), TGF-*β*, Fibroblast Growth Factor (FGF), insulin, and Insulin-like Growth Factor (IGF-1)] [113, 114]. The signal transduction system following membrane activation is played by guanine nucleotide regulatory proteins, phosphoinositide metabolism, cyclic nucleotides (cAMP and cGMP), and calcium [113, 114]. 

Different agonists modulate VSMC responses by activating tyrosine kinases through receptor and nonreceptor mechanisms. In particular, IGF-1 through its specific receptor leads to direct activation of extracellular signal regulated kinases (ERK 1/2), whereas Ang II and other mediators activate tyrosine kinase pathway by indirect mechanisms such as increased hydrogen peroxyde (H_2_O_2_) production mediated by NADPH oxidase [113, 114].

 Furthermore, VSMC which exhibit functional insulin receptors able to activate the classical signaling pathways—that is, Insulin Receptor Substrates/phosphatidylinositol 3-kinase (IRS/PI3-K) pathway and Mitogen-Activated Protein Kinase (MAPK) pathway [115–118], are targets of insulin action [115]. Through the PI3-K pathway, insulin stimulates glucose transport, induces the well-differentiated contractile state, antagonizes the effects of PDGF and increases NO production through NO synthase (NOS) activation [22, 78, 115, 116]; in the presence of PI3-K inhibitors, insulin, via the MAPK pathway, influences chemotaxis [117], DNA synthesis and proliferation [118–120]. 

In our previous studies in cultured human and rat arterial VSMC, we observed that insulin, through NOS activation with PI3-K-dependent mechanism, elicits a concentration-dependent increase of cGMP levels, increases cAMP content, and enhances the effects of the PGI_2_ analogue Iloprost, of *β* -adrenoceptor agonists and of forskolin on cAMP levels [22, 78, 84]. Insulin attenuates also the agonist-induced increase of intracellular calcium by inhibiting the inositol-triphosphate sensitive calcium release from intracellular stores and by some other biochemical mechanisms mediated by the PI3-K pathway [22, 78, 84, 115]. 

Furthermore, insulin can influence the hemodynamic balance also through the synthesis and secretion of vasoconstrictive agents such as ET-1 [22, 78, 84, 115].

 Finally, via the cooperation of the two pathways, insulin activates the Hypoxia Inducible Factor (HIF)/Vascular Endothelial Growth Factor (VEGF) pathway [22, 78, 121, 122]. HIF-1 represents a “master switch” protein generated in response to hypoxia, able to influence erythropoiesis, vasomotion, glucose metabolism, cell proliferation and survival, iron metabolism and angiogenesis; in normoxic conditions, the HIF-1 system is also induced by cytokines and growth factors [123, 124]. VEGF is a mitogen and a survival factor for endothelial cells, able to induce vascular permeability and to regulate physiological and pathological angiogenesis, with a particular role in the postischemic revascularization [125].

### 4.2. Vascular Smooth Muscle Cells in the Pathogenesis of Atherosclerosis

Following repeated or chronic arterial wall injury, such as in arterial hypertension and exposure to other cardiovascular risk factors, VSMC respond by migration into the intima, secretion, as well as by increased proliferation [38, 126, 127].

VSMC migration to the intima from the media depends on mechanisms regulated by soluble growth factors/chemoattractants, as well as by interactions with extracellular matrix [38, 126, 127]. The secretion process increases extracellular matrix formation as well as the release of proteins involved both in the digestion of major components of the extracellular matrix, such as MMPs [128–130], and in angiogenesis, such as HIF-1 and VEGF [131–134]. 

A stimulating role on VSMC is exerted by cytokines and growth factors, including IL-1*β*, IL-6, TGF-*β* 1, TNF-*α*, thrombin, bFGF, IGF-1, PDGF, urokinase plasminogen activator (u-PA), Ang II, and VEGF [38, 126, 127, 133–137]; cytokine-dependent activation, in particular, increases the synthesis of MMPs and their processing from inactive zymogens to the active enzymes [128–130, 138–141]; the same effect has been recently described for C-reactive protein (CRP) [140], and for sCD40L, which increases VSMC proliferation and migration through the MMP-9 pathway [47, 142].

Enhanced proliferation of VSMC has been considered for many years only a mechanism involved in atherosclerotic plaque formation; however, since plaques prone to rupture (the so called “unstable” plaques) show a paucity of VSMC compared with the “stable” ones [143], it has been recently recognized a beneficial role of VSMC also in plaque stabilization and therefore in one of the main mechanisms involved in the prevention of cardiovascular events which are the consequence of plaque rupture and superimposed thrombosis [144]. In recent years, the role of VSMC apoptosis within the atherosclerotic plaques [145, 146], has been considered one of the major causes of plaque rupture by thinning the fibrous cap [147, 148]. Furthermore, VSMC apoptosis has proinflammatory effects and increases macrophage infiltration through the release of IL-1*α* and the up-regulation of MCP-1 and interleukin-8 (IL-8), responsible of macrophage infiltration *in vivo* [146, 148, 149]; *in vitro* studies showed that VSMC apoptosis also promotes both thrombin generation [150], and vascular calcification [151].

Finally, through the generation and release of microparticles, apoptotic vascular cells are thrombogenic locally, thus contributing to increase the thrombogenic potential of the lipid core [152], and systemically [153]; evidences from *in vitro* studies support this effects by showing that microparticles containing TF are released by cultured VSMC in response to stimuli mimicking minimal apoptosis or flow conditions [154]. 

Migration and proliferation of VSMC are also under the inhibitory control of the nuclear receptors Peroxisome Proliferator-activated Receptor (PPAR) alpha [155, 156] and gamma [157].

### 4.3. Impairment of Vascular Smooth Muscle Cells in Insulin Resistance States and Obesity

In obese subjects several studies showed an impaired arterial vasodilation, mainly involving cerebral, mesenteric, coronary, and skeletal muscle districts [158, 159]; a pivotal role in this phenomenon is played by endothelial dysfunction related to increased secretion of proinflammatory cytokines, reduced circulating levels of adiponectin, and enhanced release of free fatty acids: all these abnormalities alter gene expression and cell signaling in vascular endothelium, cause vascular insulin resistance, modify the release of endothelium-derived factors, and increase vascular oxidative stress [158–163]. In particular, the altered pattern of adipocytokine secretion characterizing central obesity—that is, reduced adiponectin and elevated levels of leptin, resistin, TNF*α*, and IL-6—increases the production of superoxide anion (O_2_^−^), that interferes with NO availability, thus reducing vasodilation [163, 164]. 

Furthermore, insulin resistance, which characterizes human obesity and involves also the vascular effects of the hormone [115, 123, 165, 166], can determine *per se* hemodynamic consequences by impairing the balance between the vasodilating insulin actions exterted via the NO/cGMP/PKG pathway and the vasoconstricting-ones mainly exerted via ET-1 in favour of the last-ones, as extensively reviewed [22, 78, 84, 115, 123, 167].

Since VSMC from animal models of insulin resistance show alterations in mechanisms involved in vasodilation, migration, and proliferation, a role of these vascular cells in hemodynamic alterations of obesity cannot be ruled out [22].

As previously mentioned NO/cGMP/PKG pathway plays a key role in VSMC-dependent vascular responses in physiological states [110, 111]: in particular, PKG initiates several phosphorylation events leading to VSMC relaxation through a reduction of free intracellular calcium levels, a decreased sensitivity of the contractile apparatus to calcium, a rearrangement of cytoskeleton, a dephosphorylation of myosin light chain, and a phosphorylation of two filament-actin binding proteins, Vasodilatory-Stimulated Phosphoprotein (VASP) and the 20-kDa heat shock-related protein (HSP-20) [111]. PKG is also involved in VSMC proliferation and differentiation via its ability to modulate gene expression and protein synthesis [111].

Studies from our laboratory showed that VSMC from obese, insulin-resistant Zucker fa/fa rats—the classical animal model of insulin resistance due to defects in leptin receptors—show a reduced insulin ability to increase NO synthesis [121], and a reduced response to the NO/cGMP/PKG pathway [168]. In particular, VSMC from obese Zucker fa/fa rats shows: (i) baseline higher cGMP concentrations due to a reduced catabolism by phosphodiesterases; (ii) impairment of the NO ability to increase cGMP by activating the soluble guanylate cyclase; (iii) reduction of the NO and cGMP ability to activate PKG, as mirrored by a reduced ability to phosphorylate VASP at serine 239 and to activate phosphodiesterase 5 [168]. Interestingly, we also observed that VSMC from obese insulin-resistant Zucker fa/fa rats also show higher levels of O_2_^−^, and that antioxidants prevent the multiple defects of the NO/cGMP/PKG pathway, whereas H_2_O_2 _ reproduces these defects in VSMC from lean, insulin sensitive Zucker fa/+ rats [168]: these data support the pivotal role of oxidative stress in the reduced response to NO in this animal model of obesity and insulin resistance, and can explain the reduced endothelium-independent relaxation observed by some authors in these animals *in vivo* [169].

Other investigations in animal models showed that increased proliferation and migration of VSMC are a main feature accounting for atherogenic arterial lesions in insulin resistant states [170, 171]. In a rat model of obesity and type 2 diabetes mellitus, it has been described both a numerical increase and functional abnormalities in intimal VSMC and the occurrence of VSMC accumulation in atherosclerotic lesions, with a direct correlation between VSMC proliferation and insulin concentrations [170, 172, 173]; the involvement of endogenous cytokines (especially TNF-*α*), and the receptors of advanced glycation end products (AGE) [RAGE] in neointimal formation in obese Zucker rats has been recently recognized [174].

These observations are clinically relevant for the insulin resistant patients who undergo revascularization procedures in coronary arteries and in other vascular districts [175–177], since VSMC migration and proliferation lead to excessive neointima formation, which is the primary mechanism responsible of restenosis [176]. It has also been observed that in patients with type 2 diabetes the compensatory hyperinsulinemia associated with insulin resistance strongly predicts neointimal VSMC proliferation [178], and that insulin resistance and endothelial dysfunction are independent predictors of early restenosis after coronary stenting in humans [179].

## 5. Influence of Adipocytokines on Platelets and Vascular Smooth Muscle Cells in Obesity

As previously mentioned, the altered pattern of adipocyte-derived hormones and cytokines is deeply involved in the chronic proinflammatory state characterizing patients with central obesity and partially accounts for their increased cardiovascular risk [5–7, 9–11, 15, 18].

Changes in adipocyte-derived factors enhance oxidative stress by activating oxidases, interfere with NO availability and influence cell proliferation and apoptosis [91, 171, 180, 181].

Several evidences indicated that adipokines and cytokines influence platelet production and responses and VSMC function. In this part of the paper we will focus on the effects of the different adipocytokines on these cells deeply involved in atherogenesis and atherothrombosis.

### 5.1. Interleukin-6 (IL-6)

 Interleukin-6 (IL-6) is a multifunctional proinflammatory cytokine produced by different cell types, including those present in adipose tissue [174, 182]: adipose tissue, in particular, contributes to up to 35% of circulating IL-6 levels [174].

In healthy individuals IL-6 expression is due to a tight regulation dependent on a complex hormonal network related to glucocorticoid and catecholamine secretion [182, 183].

Increased IL-6 expression and circulating levels have been associated with a variety of diseases, including metabolic and vascular diseases, such as central obesity, the metabolic syndrome, type 2 diabetes mellitus, atherosclerosis and, in particular, atherosclerotic coronary artery disease [182, 183].

IL-6—together with other cytokines—is a key risk factor for the development of atherothrombotic diseases due to its effect in plaque development and destabilization via release of other proinflammatory cytokines, oxidation of lipoproteins by phospholipases, stimulation of acute phase protein secretion, release of prothrombotic mediators, and activation of MMPs [183, 184]. Moreover, the increased ROS formation by vascular enzyme systems under proinflammatory conditions may play a critical role in the cross talk between IL-6 and other vasoactive substances, such as Ang II and catecholamines [185–187].

#### 5.1.1. IL-6 and Platelets

As far as megakaryocytopoiesis is concerned, IL-6 acts synergistically with thrombopoietin (TPO), other interleukins, and growth factors in promoting the maturation of megakaryocyte precursors [188–192]; actually, *in vivo *administration of IL-6 to humans increases circulating platelet counts [190, 192]. This effect may explain the association between the increased markers of chronic inflammation and the elevated platelet count in obese women [193]: this phenomenon has been considered prothrombotic, since higher platelet counts are associated with adverse clinical outcomes in patients with acute coronary events [194].

**Figure 4:**
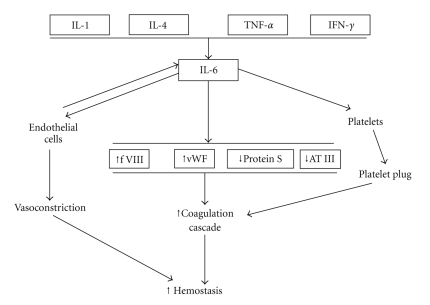
Mechanisms involved in the alterations of the coagulative balance induced by interleukin-6 (IL-6). IL-1, interleukin-1; IL-4, interleukin-4; TNF-*α*, tumor necrosis factor-*α*; IFN-*γ*, interferon-*γ*; f VIII, factor VIII, vWF, von Willebrand factor; AT III, antithrombin III.

As summarized in Figure 4, IL-6 is responsible of an acute prothrombotic state [184, 195] through mechanisms involving: (a) increased expression of TF, fibrinogen, factor VIII and vWF; (b) activation of endothelial cells; (c) reduced levels of hemostasis inhibitors, such as antithrombin and protein S [195]. Furthermore, IL-6 influences platelet function by enhancing thrombin-induced activation [195], and modulates platelet responses by increasing ROS production [174].

#### 5.1.2. IL-6 and VSMC

IL-6 influences both vessel wall cells and their progenitors; beyond its effects on resident endothelial cells, IL-6 exerts pro-angiogenic actions by stimulating migration and proliferation of circulating endothelial progenitors [196]. 

As far as VSMC are concerned, proinflammatory mediators increase in these cells synthesis and release of IL-6 [197]. Additionally, IL-6 exerts specific effects on VSMC: in particular, it is involved in growth factor-dependent VSMC migration, by interplaying with VEGF and TNF-*α* [198–200], and stimulates VSMC proliferation with PDGF-dependent and -independent mechanisms [201, 202]. 

These effects explain the clinical observation that increased IL-6 levels both in coronary and systemic circulation are a risk factor for restenosis after angioplasty [203, 204].

### 5.2. Tumor Necrosis Factor-*α* (TNF-*α*)

TNF-*α* is a pleiotropic, proinflammatory cytokine, produced as 17 kDa protein and secreted as a 51 kDa trimer by a variety of cells including not only adipose tissue (see below) but also macrophages, natural killer cells, T-cells, endothelial cells and VSMC [205]; its presence has been also recognized in human atheroma [206]. 

Adipose tissue has been identified as one of the main sources of TNF-*α*: the majority of TNF-*α* produced in adipose tissue is derived from infiltrating macrophages and not from mature adipocytes [8, 13–15, 207].

 Several studies evidenced an increased TNF-*α* circulating level both in obese nondiabetic subjects and in type 2 diabetic patients [13, 207–209] and hypothesized its involvement in the pathogenesis of obesity-linked insulin resistance [209–212].

As far as the TNF-*α* effects on vascular function are concerned, it has been observed that acute administration of TNF-*α* exerts *per se* a vasodilatory effect, but impairs endothelium-dependent vasodilation in response to insulin and acetylcholine in healthy humans [212–214]; furthermore, it inhibits the vasodilating actions of insulin in vessels of rat skeletal muscle [213–215]; the TNF-*α* interference with endothelial-mediated vasodilation is also due to shortening the half-life of eNOS mRNA in endothelial cells [215, 216] and to the increase of ET-1 synthesis and spillower [216].

TNF-*α* induces inflammatory changes in vessel wall by activating the transcription factor Nuclear factor-*κ* *β* (NF-*κ* B) [217], which increases the expression of ICAM-1 and VCAM-1, and the production of MCP-1 and M-CSF from endothelial cells and VSMC. 

Plasma TNF-*α* concentrations predict vascular damage, since they are associated with early atherosclerosis in middle-aged healthy men [218]; furthermore, elevations of TNF-*α* in the stable phase after myocardial infarction were associated with an increased risk of recurrent coronary events [219].

#### 5.2.1. TNF-*α* and Platelets

*In vitro* studies showed that TNF-*α* promotes platelet aggregation [220] and ROS production mainly through activation of the arachidonic acid pathway [221, 222]. 

Other data indicate that TNF-*α* influences platelet function also by increasing the secretion of leptin, which acts as proaggregating hormone.

At present, the results from *in vivo* studies are not conclusive and there are not evidences to identify TNF-*α* as a prothrombotic or antithrombotic factor in human patients [223].

#### 5.2.2. TNF-*α* and VSMC

 Human VSMC are both source and target of TNF-*α* [205] which, together with interferon-*γ*and IL-1, stimulates IL-6 production by this cell type [224]. Through NF-*κ* B pathway, TNF-*α* increases synthesis/release of MMP from VSMC [217]. Thus, TNF-*α* is deeply involved in plaque inflammation and instabilization also by the mechanisms involving VSMC as described in the first part of this review.

### 5.3. Leptin

Leptin is a 167-amino acid adipokine, primarily synthesized and released by mature adipocytes, although expressed also in many other tissues including muscle, placenta and gastric epithelium. Its circulating levels are highly correlated with BMI [225].

Leptin receptors have been identified both in the hypothalamus and in extrahypothalamic tissues and its main role is to inform the brain regarding the amount of stored fat, thus primarily regulating food intake and energy expenditure [226]; however, in obese humans increased leptin levels are unable to induce weight loss: this phenomenon is attributed to a selective resistance to its metabolic actions [227].

Leptin, which has a structural and functional relation to proinflammatory cytokines such as IL-6, also influences angiogenesis, inflammation, arterial blood pressure and secretion of other adipokines [10, 180, 181, 207].

 In animal models, chronic hyperleptinemia is involved in oxidative stress by decreasing plasma levels of the antioxidant enzyme paraoxonase-1, an activity linked to circulating lipoproteins [228]. This leptin effect is followed by increased plasma and urinary concentration of isoprostanes reflecting an increased oxidative stress [228]. Evidences for an involvement of leptin in atherosclerosis have been recently provided by direct leptin administration in apolipoprotein deficient mice [229] and by the finding that ob/ob mice which lacked functioning leptin gene are resistant to atherosclerosis despite the presence of obesity and diabetes [230]. Also in humans, the vascular actions of leptin are considered proatherogenic and the increase of its circulating levels due to adiposity has been involved in the pathogenesis of vascular damage [231].

At present, clinical investigations considered leptin as an independent risk factor for cardiovascular [232–234] and cerebrovascular diseases [235, 236], evidencing that its plasma concentrations are independently associated with the intima-media thickness of the common carotid artery [237], and with the degree of coronary artery calcification in patients with type 2 diabetes mellitus, after controlling for adiposity and CRP [238, 239]; furthermore, hyperleptinemia could be involved in the increased risk of postangioplasty restenosis [179, 240]. 

#### 5.3.1. Leptin and Platelets

The pro-thrombotic actions of leptin *in vivo* are related to an influence on platelet function, and on coagulation/fibrinolysis balance, resulting in enhanced agonist-induced platelet aggregation and increased stability of arterial thrombi [241–243]. 

The long form of the leptin receptor (Ob-Rb) is present in human platelets and can be related to platelet activation by a specific pathway downstream of leptin-induced Janus kinase 2 (JAK2) activation including PI3-K and phospholipases C*γ*_2_ and A_2_, which influence cAMP hydrolysis, GPIIb/IIIa expression, and thromboxane synthesis [241]: these findings induced to consider circulating plaletets as a major target of leptin action, suggesting a possible direct link between obesity and thrombotic complication [241, 242].

Studies *in vitro* showed that leptin synergizes with subthreshold concentrations of agonists—such as ADP and thrombin—to induce platelet aggregation [242–245], but is unable to directly aggregate platelets. The involvement of leptin in the increased platelet activation in human obesity is not universally accepted, since recent studies provided conflicting results about platelet responsiveness to leptin in overweight and obesity [246, 247]; actually, in one study the effect of leptin on ADP-induced platelet aggregation is attenuated in obese individuals due to the receptor desensitization [245], whereas different results have been provided in another report [247].

Normal weight subjects undergoing complete caloric deprivation have an increased sensitivity of hemostatic responses to leptin [248]: this phenomenon indicates that the sensitivity of leptin receptors on platelet membranes is influenced by body composition [248]. In light of this, the resistance to leptin in overweight or obese patients could represent a protective mechanism against the excess pro-thrombotic stimulation produced by obesity-related hyperleptinemia [249].

However, other mechanisms by which leptin may contribute to vascular damage—such as inflammation, oxidative stress, endothelial dysfunction, as well as increased sympathetic tone—are preserved in obese subjects [240, 250–252] and can contribute to pro-thrombotic action of leptin [240, 253].

#### 5.3.2. Leptin and VSMC

Leptin exerts hemodynamic actions, even though its effects on the arterial wall have not been fully elucidated at present. Evidences in animals and humans showed that leptin administration induces acute vasodilation in different vascular districts, including human coronary arteries [207, 254, 255].

The involvement of a receptor-mediated NO release from endothelium has been shown in several, but not all, experimental models of leptin-induced vasodilation [255, 256]; despite the increase of NO release, acute leptin administration induced blood pressure reduction only in sympathectomized animals, likely for a compensatory activation of the sympathetic nervous system [256, 257]. Insulin interacts with leptin to modulate vascular responses, mainly by enhancing NO production by endothelium [258].

Due to the presence of active receptors in VSMC [259], leptin induces VSMC migration, proliferation, and expression of MMP-2, as shown in human aorta *in vitro* [260]; furthermore, *in vitro* studies showed that leptin stimulates osteoblastic differentiation of VSMC and hydroxyapatite production, thus explaining the relationships between circulating levels of leptin and the degree of coronary artery calcification [238, 239, 261].

Indirect mechanisms responsible of other leptin effects on VSMC are referred to oxidative stress which can cause endothelial or VSMC damage and stimulation of low-grade vascular inflammation [262].

### 5.4. Adiponectin

Adiponectin is a 30-kDa protein collagen-like molecule that shares substantial homology with subunits of complement factor C1q [207, 263, 264]. It is expressed almost exclusively in mature adipocytes where is the most abundant adipokine synthesized and released locally and in the blood stream. It accounts for 0.01% of the total plasma proteins in form of trimer, hexamer and high molecular weight 12-to 18-mer [207, 263, 264]. Adiponectin synthesis is detectable also in human and murine cardiomyocytes [265].

As opposite to the other adipokines, circulating adiponectin is negatively related to the increase of fat mass likely owing to the abnormal hormonal milieu mainly caused by the inhibitory effects exerted by the increased local TNF-*α* levels, by the oxidative stress and by the proinflammatory state which prevail in central obesity [207, 264, 266, 267]; its secretion is restored as a consequence of weight loss [267].

Adiponectin exerts insulin-sensitizing effects by increasing glucose uptake, NO production, and free fatty acid oxidation [268–270] and shows an antiinflammatory activity mainly through a cAMP-mediated interference with NF-*κ* B signaling [271]. 

In vascular wall, the antiinflammatory properties of adiponectin—which account for its antiatherogenic effects—reduce cell expression of adhesion molecules and scavenger receptors [272, 273]. This phenomenon is mediated by the inhibition of the effects of TNF-*α* and Ang II on both endothelial cells (expression of adhesion molecules, protection, increase of permeability, production of ROS) and macrophages (decrease of cytokine production mediated by NF-*κ* B signaling) [268, 271, 272]. 

The vasculo-protective effects of adiponectin have been recently confirmed in clinical studies, by showing that its decreased levels contribute to the metabolic and vascular abnormalities in obese subjects [264, 268, 274].

#### 5.4.1. Adiponectin and Platelets

In animals adiponectin plays a role as antithrombotic factor [275]. Actually, there is an accelerated thrombus formation after carotid arterial injury in adiponectin knockout mice in comparison to wild-type ones: the potential involvement of platelets in this effect is suggested by the presence of active adiponectin receptors AdipoR1 and AdipoR2 in wild-type mice and by the enhanced platelet response to ADP and collagen in adiponectin knockout animals [275]. The same receptors are present in isolated human platelets and in human megakaryocytic cell lines; in humans, however, adiponectin does not influence platelet activation by ADP and collagen [276].

It has been hypothesized that adiponectin exerts indirect anti-thrombotic effects by decreasing the circulating concentrations of TNF-*α* and IL-6 and by interfering with their pro-thrombotic activities, mainly dependent on increased oxidative stress and decreased NO bioavailability [20, 277, 278].

#### 5.4.2. Adiponectin and VSMC

Adiponectin suppresses proliferation and migration of smooth muscle cells by directly binding to several growth factors, particularly PDGF BB, FGF, and heparin-binding epidermal growth factor-like growth factor (HB-EGF) [279, 280].

Following experimental vascular injury, immunohistochemical analysis shows the presence of adiponectin in the walls of the catheter-injured vessels but not in intact vascular walls [281]; thus, it can be hypothesized that adiponectin secreted from adipose tissue reaches the injured arteries after the lesion of the endothelial barrier and accumulates in vascular walls, thus reducing the atherogenic process [281].

Finally, adiponectin may favor plaque stabilization decreasing MMP activity by modulating the expression of the Tissue Inhibitor of Metalloproteinase-1 (TIMP-1) through increased IL-10 secretion [282].

### 5.5. Ghrelin

Ghrelin is a 28-amino acid peptide hormone, predominantly produced by the stomach [283], which acts as a natural ligand of the Growth Hormone (GH) secretagogue (GHS) receptor type 1a (GHS-R1a), and plays a role in the central control of appetite together with leptin [284]. Circulating ghrelin levels are inversely related with degree of obesity evaluated by BMI [285].

More recently, it has been demonstrated that ghrelin also influences the cardiovascular system, by decreasing sympathetic activity and producing vasodilation through an endothelium-independent mechanism [286].

Furthermore, ghrelin exerts antiinflammatory effects in vessels, improves left ventricular function and shows antiapoptotic actions on cardiomyocytes [286].

It has been suggested that the decrease of ghrelin in obese patients is involved in their enhanced cardiovascular risk and that ghrelin administration exerts protective effects in patients with obesity-related metabolic syndrome [287]. 

#### 5.5.1. Ghrelin and Platelets

The protective cardiovascular effects of ghrelin likely do not involve platelets, since *in vitro* this hormone does not affect platelet aggregation or adhesion [276].

#### 5.5.2. Ghrelin and VSMC

VSMC are targets of ghrelin, which inhibits *in vitro* Ang II-induced proliferation and contraction in a dose-response manner via the cAMP/PKA pathway [288], and prevents vascular calcification in rats [289].

### 5.6. Apelin

Apelin is an endogenous peptide ligand for the orphan G-coupled APJ receptor, which exhibits close homology with the angiotensin-like 1 receptor [290]. It exists at least in three molecular forms, consisting of 13, 17, or 36 amino acids, all originating from a common 77-amino acid precursor, and is present in several tissues, primarily in the vascular endothelium [290]. Recently, apelin has been identified in adipose tissue [291], where its gene expression is increased by insulin, steroids and TNF-*α* [292, 293]. Its synthesis and release are up-regulated in obesity, whereas its expression in adipose tissue and its circulating levels are reduced by weight loss [292].

Apelin is mainly considered a vascular hormone: actually, it induces an endothelium-dependent vasodilation [294, 295], exerts inotropic effects [296], and plays a protective role in the pathogenesis of heart failure by modulating the harmful effects of Ang II [294, 295].

 Apelin is down-regulated in patients with heart failure and up-regulated by a favourable left ventricular remodeling [295]. 

The role of apelin-APJ system in atherogenesis is controversial. Some apelin actions—including modulation of endothelial oxidative stress and macrophage activation, NO-dependent vasorelaxation and reduction of arterial blood pressure [295, 297, 298]—are clearly protective, whereas other effects on arterial vessels might be detrimental by favouring the atherogenic damage [299].

#### 5.6.1. Apelin and VSMC

In the vascular system, the apelin-APJ system is expressed in both endothelium—where it is mitogenic [300]—and in VSMC [301]. Acute apelin administration in man causes NO-mediated arteriolar vasodilation [294]. At present, data about the direct effects of apelin on VSMC are incomplete; it has been suggested an influence on myosin light chain phosphorylation via APJ receptors, responsible of vasoconstriction [302], and a proliferative effect on VSMC, induced by oxidative stress [299, 303].

### 5.7. Visfatin

Visfatin, previously recognized as a protein involved in immune B-cell maturation [pre-B cell colony-enhancing factor (PBEF)] [304], is abundantly expressed in visceral adipose tissue and is up-regulated in some, but not in all, the animal models of obesity [305]. Preliminary studies suggest that plasma visfatin concentrations are increased in humans affected by abdominal obesity and type 2 diabetes mellitus [306, 307].

**Figure 5:**
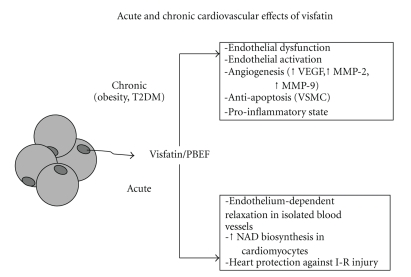
Acute and chronic cardiovascular effects of visfatin. VEGF, vascular endothelial growth factor; MMP-2, matrix metalloproteinase-2; MMP-9, matrix metalloproteinase-9; PBEF, pre-B cell colony-enhancing factor I-R, ischemia-reperfusion.

Although it is not a cytokine, visfatin expression is up-regulated by cytokines: several studies showed its involvement in metabolic and vascular homeostasis [308, 309]. Metabolic insulinomimetic effects, characterized by reduction of hepatic glucose production and stimulation of peripheral glucose utilization, has been attributed to binding to insulin receptor at a site different from that of insulin [308], even though recent results excluded a visfatin-dependent direct activation of insulin signaling pathway and attributed its action to nicotinamide phosphoribosyltransferase activity (Nampt) [310]. Vascular effects are both chronic and acute (see Figure 5): chronic exposure to high visfatin concentrations—such as in obesity and in type 2 diabetes mellitus—promotes endothelial dysfunction, angiogenesis and atherosclerotic plaque instabilization, whereas acute visfatin administration stimulates eNOS expression and activity in endothelial cells [309] and directly protects cardiomyocytes against the detrimental effects of acute ischemia-reperfusion injury [311].

#### 5.7.1. Visfatin and VSMC

Visfatin influences VSMC phenotype maturation from a proliferative noncontractile to a nonproliferative contractile one required for vasomotor function [312], and promotes VSMC proliferation in perivascular adipose tissue by a paracrine mechanism [313].

### 5.8. Resistin

Resistin has been originally identified as an adipocyte-secreted peptide able to induce insulin resistance in rodents; in humans it is represented by a 12.5 kDa cysteine-rich protein of 108 amino acids [314]. Also in humans resistin is produced by adipose tissue and may act both in paracrine and in endocrine fashion [314]: however, in contrast to mice, only a low level of expression of resistin has been found in mature adipocytes in humans [315, 316]. Furthermore, resistin expression has been demonstrated in bone marrow, trophoblastic cells of placenta, pancreas, synovial tissue, and circulating blood cells [317].

Resistin is an important regulator of glucose homeostasis, adipogenesis, and, potentially, inflammation [317]; in particular, it can induce insulin resistance by regulating adipose tissue deposition through a negative feedback mechanism [317], and exerts proinflammatory effects through activation of the transcription factor NF-*κ* B [318].

**Figure 6:**
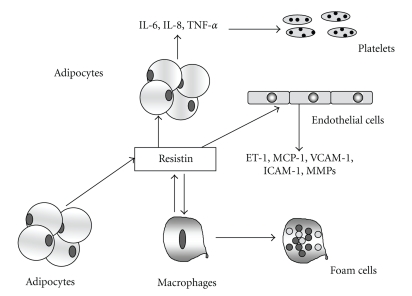
Potential vascular effects of resistin. IL-6, interleukin-6; IL-8, interleukin-8; TNF-*α*, tumor necrosis factor-*α*; ET-1, endothelin-1; MCP-1, monocyte chemoattractant protein-1; VCAM-1, Vascular Cell Adhesion Molecule-1; ICAM-1, Intercellular Adhesion Molecule-1; MMPs, matrix metalloproteinases.

The interplay between resistin and vascular wall cells can potentially contribute to the development of atherosclerotic lesions (Figure 6): in particular, it favors angiogenesis by inducing endothelial cell growth activation and migration, mainly by increasing ET-1 release [319, 320], and potentiating the effect of CD40L [321]; furthermore, it is involved in lipid storage in macrophages [317, 322].

#### 5.8.1. Resistin and VSMC

Resistin induces proliferation of cultured human aortic VSMC through both ERK 1/2 and Akt signaling pathways [323]. Furthermore, hypoxia increases resistin expression in cultured rat VSMC [323].

### 5.9. Endothelin

Increased circulating levels of ET-1 have been observed in patients affected by central obesity and metabolic syndrome [324]. ET-1 elevation is proportional to hyperinsulinemia [324, 325], and weight loss by diet intervention reduces both serum insulin and ET-1 [326]. 

The increase of ET-1 accounts for a prevailing vasoconstrictive effect of insulin in insulin resistant states, in which the insulin-induced, PI3-K-mediated increase of NO is impaired [325]; furthermore, ET-1 contributes *per se* to vasoconstriction by influencing calcium fluxes, by activating the renin-angiotensin system, and by inducing VSMC hypertrophy [327].

#### 5.9.1. ET-1 and Platelets

Platelets are a potential target of circulating ET-1. However, as recently reviewed, ET-1 effects on platelets are still conflicting [328]: some studies showed that *in vitro* exposure to ET-1 induces platelet activation or increases platelet responses to aggregating agonists [327–329], other studies, however, failed to detect any direct effect [329] or even showed a decrease in platelet responses [328].

These conflicting results may be due to complex interactions between platelet ET(A) and ET(B) receptors.

#### 5.9.2. ET-1 and VSMC

VSMC are both a source and a target of ET-1 [330–333]. As recently reviewed [333], ET-1 stimulates VSMC proliferation [334], migration [335], contraction [336], extracellular matrix synthesis and remodeling [337, 338], and expression of other proatherogenic growth factors such as PDGF and TGF-*β* [339].

## 6. Conclusions

The major adverse consequences of central obesity are related to the development of type 2 diabetes mellitus and of athero-thrombotic vascular diseases, which account for a high disease-related mortality [1, 5–7]. As extensively confirmed, the main alterations of central obesity involved in vascular damage are recognized in the impaired systemic metabolic homeostasis as well as in the presence of an active low-grade chronic inflammatory process in tissues that are relevant for metabolism, such as adipose tissue, liver, muscle and arterial wall [6, 11, 13, 15, 19, 340].

In particular, the activation of an inflammatory process by adipose tissue is related to impairment of the secretion pattern of adipocytokines, including increased local availability of major cytokines [6, 7, 13, 14, 18, 340, 341], as well as enhanced synthesis and secretion of proinflammatory adipokines [13–15] and reduced availability of protective, insulin sensitizing peptides, including adiponectin and ghrelin [2, 15, 340–343].

On the basis of the large number of reviewed studies, a relevant role can be recognized to impairment of platelet and VSMC functions in the pro-thrombotic tendency, proinflammatory state and accelerated atherogenesis of the patients with central obesity [20–22]. 

Impaired synthesis and/or secretion of single adipocytokines which can interplay with platelets and VSMC are deeply involved in these phenomena.

As extensively reviewed, a large body of evidences showed detrimental actions of the increased synthesis and secretion of several mediators which act through pro-thrombotic and proliferative actions and oxidative stress, including TNF-*α*, interleukins and likely leptin [342]. However, also the lack of the protective effects of adipokines such as adiponectin and ghrelin has to be considered another relevant feature of the proatherogenic milieu characterizing the altered endocrine pattern of the patients with central obesity. In particular, adiponectin is a relevant anti-thrombotic adipokine, as widely reviewed [266, 268, 343] and emerging evidences underline ghrelin protective effects on insulin resistance, cardiovascular system, oxidative stress, and, likely, hemostatic balance [20, 104, 105, 343]: therefore, the reduced levels of this peptide in central obesity may be another deterimental feature increasing cardiovascular risk in obese subjects.

In summary, available evidences allow to hypothesize the presence of a complex scenario related to increased atherogenic and atherothrombotic risk in central obesity: in this context an impaired pattern of adipocytokine synthesis and secretion, rather than alteration of single mediators, has to be considered a major mechanistic link.

## Review Strategy and Selection Criteria

Searches for original articles and reviews from 1985 to February 2010 focusing on obesity, hemostasis, vascular function and adipokines were performed in MEDLINE and PubMed electronic databases. The search terms were: “adipocytokines”, “adipokines”, “adiponectin”, “apelin”, “cytokines”, “central obesity”, “endothelin-1”, “ghrelin”, “insulin resistance”, “interleukin-6”, “leptin”, “metabolic syndrome”, “obesity”, “overweight”, “platelets”, “platelet dysfunction”, “resistin”, “thrombosis”, “Tumor necrosis factor-*α* ”, “vascular smooth muscle cells”, and “visfatin”. All papers identified were English-language, full-text papers and were selected on the basis of relevance and novelty; a priority was given to those published in peer-reviewed journals.

## Abbreviations

ADP: Adenosine 5-diphosphate
AGE: Advanced glycation end products
Akt/PKB: Akt/protein kinase B
Ang II: Angiotensin II
BMI: Body mass index
cAMP: 3′, 5′ -cyclic adenosine monophosphate
CD40L: CD40 ligand
cGMP: 3',5'-cyclic guanosine monophosphate
COX-1: Cyclo-oxygenase-1
CRP: C-reactive protein
DAG: Diacylcyglycerol
EGF: Epidermal Growth Factor
eNOS: Endothelial NO synthase
ERK 1/2: Extracellular signal regulated kinases
ET-1: Endothelin-1
FACoA: Fatty acid coenzyme A
FFA: Free fatty acids
FGF: Fibroblast Growth Factor
H_2_O_2_: Hydrogen peroxide
HSP-20: 20-kDa heat shock-related protein
HSP-90: Heat shock protein-90
HIF-1: Hypoxia Inducible Factor-1
ICAM-1: Intercellular Adhesion Molecule-1
IGF-1: Insulin-like Growth Factor-1
IL-1:*β*: Interleukin-1*β*
IL-6: Interleukin-6
IP3: Inositol, 1,4,5-triphosphate
IRS: Insulin Receptor Substrates
8-iso-PGF_2*α*_: 8-iso-prostaglandin-F_2*α*_
LDL: Low density lipoproteins
MAPK: Mitogen-Activated Protein Kinase
MCP-1: Monocyte Chemoattractant Protein-1
MIF: Macrophage migration inhibitory factor
MMPs: Matrix metalloproteinases
MPV: Mean platelet volume
NF-B*κ*: Nuclear Factor-*κ* B
NO: Nitric oxide
O_2_^−^: Superoxide anion
PAF: Platelet activating factor
PAI-1: Plasminogen Activator Inhibitor-1
PBEF: Pre-B cell colony-enhancing factor
PDGF: Platelet-derived growth factor
PF-4: Platelet factor-4
PGI: Prostacyclin
PI3-K: Phosphatidylinositol 3-kinase
PIP: Phosphatidylinositol 4,5-bisphosphate
PKC: Protein kinase C
PKG: cGMP-dependent protein kinase
PPAR: Peroxisome proliferator-activated receptor
RAGE: Receptors of advanced glycation end products (AGE)
RANTES: Regulated on activation, normal T-cell expressed and secreted
ROS: Reactive oxygen species
sGC: Soluble guanylate cyclase
TF: Tissue factor
TGF-*β*: Transforming growth factor-*β*
TIMP-1: Tissue Inhibitor of Metalloproteinase-1
TNF-*α*: Tumor necrosis factor-*α*
TPO: Thrombopoietin
TXA: Thromboxane A_2_
u-PA: Urokinase plasminogen activator
VASP: Vasodilatory-Stimulated Phosphoprotein
VCAM-1: Vascular Cell Adhesion Molecule-1
VEGF: Vascular Endothelial Growth Factor
VIP: Vasoactive Intestinal Polypeptide
VSMC: Vascular smooth muscle cells
vWF: Von Willebrand factor.
